# Endovascular Thrombectomy for Acute Ischemic Stroke due to Calcified Cerebral Emboli

**DOI:** 10.1155/srat/5538938

**Published:** 2025-05-21

**Authors:** Hannes Schacht, Peter Schramm, Björn Machner, Björn-Hergen Laabs, Philipp J. Koch, Ulf Jensen-Kondering, Alexander Neumann

**Affiliations:** ^1^Department of Neuroradiology, University Medical Center Schleswig-Holstein, Lübeck, Germany; ^2^Department of Neurology, University Medical Center Schleswig-Holstein, Lübeck, Germany; ^3^Department of Neurology, Schoen Clinic Neustadt, Neustadt in Holstein, Germany; ^4^Institute of Medical Biometry and Statistics (I. R. K.), University Medical Center Schleswig-Holstein, Lübeck, Germany

## Abstract

**Background:** Calcified cerebral emboli (CCEs) represent a rare cause of acute ischemic stroke and can pose technical challenges for neurointerventionalists. The few studies on endovascular thrombectomy (EVT) of CCE to date show poor recanalization rates and unfavorable outcomes.

**Objective:** This study is aimed at investigating the technical and clinical results concerning EVT of CCE compared with noncalcified cerebral emboli (NCCEs).

**Methods:** All cases of EVT for acute stroke from January 2014 to December 2021 from a single center were analyzed retrospectively. Emboli with a maximum density of ≥ 130 Hounsfield units on preinterventional CT scans were considered calcified. Propensity score matching was performed to compare technical and clinical results between patients with CCE and NCCE.

**Results:** CCEs were present in 26 of 1004 cases (2.6%). Successful recanalization (mTICI ≥ 2b) was achieved less frequently in CCE (CCE: 62%, NCCE: 92%, *p* = 0.009). Also, first-pass reperfusion was less common in CCE (CCE: 12%, NCCE: 46%, *p* = 0.006). In CCE, infarct growth was more frequent (CCE: 81%, NCCE: 42%, *p* = 0.004) and more severe (*p* = 0.005). National Institutes of Health Stroke Scale improvement after EVT was lower in CCE patients (CCE: median 2, range −23 to 20, interquartile range (IQR) 2.75; NCCE: median 5, range −8 to 17, IQR 11, *p* = 0.008).

**Conclusion:** First-pass reperfusion is less common in EVT of CCE. Also, there is a more frequent and severe infarct growth in CCE patients after EVT, which helps to understand the poorer clinical results. Thrombectomy devices optimized for CCE are desirable to improve outcomes in this subgroup of stroke patients.

## 1. Introduction

Endovascular thrombectomy (EVT) has become the standard treatment for acute ischemic stroke due to large vessel occlusion with high recanalization rates [1, 2]. In this context, first-pass reperfusion (FPR) was found to be associated with higher rates of good clinical outcome [3, 4]. In some patients, however, successful reperfusion cannot be achieved or can only be achieved after numerous maneuvers and long intervention times. The histological and mechanical properties of the emboli have been identified as factors influencing the success of EVT [5, 6]. Calcified cerebral emboli (CCEs) are a rare cause of large vessel occlusion stroke and are considerably stiffer than noncalcified cerebral emboli (NCCEs). This type of embolus can therefore pose high technical challenges for neurointerventionalists. Also, occlusions due to CCE can be overlooked in CT angiography because of their high density, which has been described as the “false patency sign” [7]. The few existing studies on EVT of CCE have reported comparatively poor angiographic and clinical outcomes [8–12]. Our aim was to analyze the technical and clinical success rate as well as the complications of EVT in CCE compared with NCCE.

## 2. Methods

### 2.1. Patients

In this retrospective, single-center study, we reviewed all consecutive EVT cases performed at our institution from January 2014 to December 2021. Approval of our institutional ethics committee was obtained (file reference: 2023-117). Preinterventional CT scans were reviewed for radiological embolus characterization, identification of the occluded vessel segment, and assessment of early ischemic changes, based on the Alberta Stroke Program Early CT Score (ASPECTS). Postinterventional CT scans were reviewed to assess the extent of infarction after endovascular treatment, based on the poststroke Alberta Stroke Program Early CT Score (ASPECTS-POST) [13]. Infarct growth was defined as the difference between ASPECTS and ASPECTS-POST. In cases of posterior circulation stroke, the posterior circulation Acute Stroke Prognosis Early CT Score (pc-ASPECTS) [14] was used. Cases in which preinterventional CT scans were not available were excluded. We recorded patient age in years at the time of intervention, sex, National Institutes of Health Stroke Scale (NIHSS) scores at the time of admission and discharge, embolus length, maximal embolus density in Hounsfield units (HU), modified Thrombolysis in Cerebral Infarction (mTICI) scale, groin to final mTICI time, the number of EVT maneuvers, used EVT techniques, and periprocedural complications. Moderate to severe subarachnoid hyperdensities on postinterventional CT images were considered a significant complication, as they were found to be associated with a worse functional outcome [15]. Emboli were considered calcified if the maximum density on nonenhanced CT scans was at least 130 HU, based on a study quantifying coronary artery calcification, which was also the basis of a previous work on EVT of CCE [10, 16]. For CCE patients, a control group with an equivalent number of NCCE patients was created based on a propensity score matching, taking into account age, sex, location of the embolus, and year of treatment. For comparison of technical recanalization success between the groups, angiographic results were divided into “successful” (mTICI 2b-3) and “unsuccessful” (mTICI 0-2a), following previous studies [1, 17]. The frequency of FPR was compared between the groups. Based on the study by Zaidat et al., FPR was defined as complete or near-complete reperfusion (mTICI 3 or 2c) after a single EVT maneuver [3]. The difference between NIHSS at admission and discharge was used to assess clinical recanalization success. The decrease from ASPECTS to ASPECTS-POST was used to compare infarct growth following EVT between the groups.

### 2.2. Statistical Analysis

Statistical analyses were conducted using Pearson's chi-square tests for categorical variables, Mann–Whitney *U* tests for continuous variables, along with a Kaplan–Meier analysis and a Mantel–Cox log-rank test to assess the probability of successful recanalization, all performed with SPSS (Version 29). Propensity score matching was performed with R (Version 4.3.1) and RStudio (Version 2023.06.0). *p* values < 0.05 were considered statistically significant.

## 3. Results

All continuous variables showed a non-normal distribution and are therefore presented as medians with interquartile ranges (IQRs).

### 3.1. Patient Characteristics

From January 2014 to December 2021, EVT for acute ischemic stroke was performed in 1081 cases at our institution. Preinterventional CT scans were available in 1004 of these cases. In 26 cases, CCEs were present (2.6%). Patients with CCE were older (median age 79, range 47–91, IQR 12) compared with the 978 patients with NCCE (median age 73, range 2–99, IQR 18, *p* = 0.027) and showed a female predominance (CCE: 69.2%, NCCE: 50.1%, *p* = 0.054).

### 3.2. Radiological Embolus Features

The 26 CCEs were of a shorter length (median 6 mm, range 2–29 mm, IQR 5 mm) than the 26 NCCE in the control group (median 10.5 mm, range 3–31 mm, IQR 12 mm, *p* = 0.015). The median maximum density was 341 HU in CCE (range 151–1582 HU, IQR 333 HU) and 76 HU in NCCE (range 53–89 HU, IQR 23 HU, *p* < 0.001).

### 3.3. Recanalization Techniques

The choice of EVT technique was at the discretion of the interventionalist in each case. Stent-retriever-based EVT maneuvers (SRT) were performed more often in cases with CCE (total 77, median 2, range 0–8, IQR 2.75) than in the matched cases with NCCE (total 37, median 1, range 0–4, IQR 1, *p* = 0.002). The number of direct aspiration thrombectomy (DAT) maneuvers did not differ significantly between the groups (CCE: *n* = 16, NCCE: *n* = 13, *p* = 0.925). In both groups, FPR was achieved more frequently (66.7% each) by using SRT (CCE: 1x DAT, 2x SRT vs. NCCE: 4x DAT, 8x SRT). All SRT maneuvers were performed in combination with an aspiration catheter. In all cases (DAT and SRT), aspiration was performed using a vacuum pump. In one case, an occlusion of the left middle cerebral artery occurred during carotid artery stenting due to a CCE detached from the carotid bifurcation. Rescue stenting of the middle cerebral artery was performed in this case after unsuccessful removal of the CCE.

### 3.4. Technical Treatment Success

**Figure 1:**
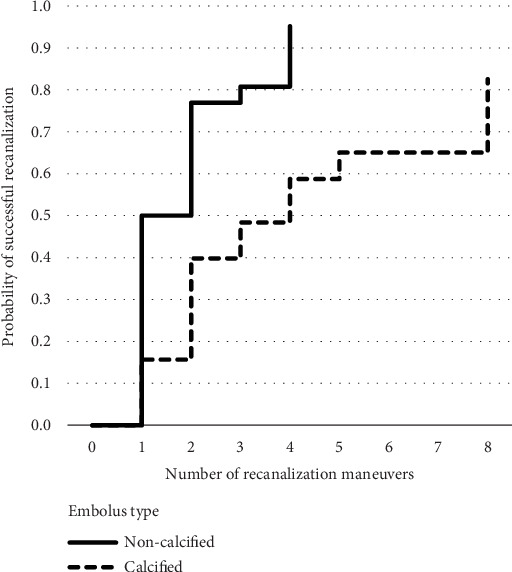
Kaplan–Meier curves displaying the probability of successful recanalization according to the embolus type.

The groin to final mTICI time was longer in the CCE group (median 77 min, range 16–230 min, IQR 62 min) compared with the control group with NCCE (median 31 min, range 11–88 min, IQR 34 min, *p* < 0.001). Successful recanalization was achieved more often in NCCE (92%) than in CCE (62%, *p* = 0.009). More recanalization maneuvers were performed in CCE (median 3, range 1–8, IQR 3) than in NCCE (median 1.5, range 1–4, IQR 1, *p* = 0.004). The number of FPR was higher in NCCE (46.2%, *n* = 12) than in CCE (11.5%, *n* = 3, *p* = 0.006). The Kaplan–Meier analysis and Mantel–Cox log-rank test showed that significantly more maneuvers were necessary to achieve successful recanalization in CCE (*p* = 0.001) (Figure 1).

### 3.5. Clinical Treatment Success

Patients with NCCE showed greater improvements in NIHSS scores (median 5, range −8 to 17, IQR 11) compared with CCE patients (median 2, range −23 to 20, IQR 2.75, *p* = 0.008).

### 3.6. Infarct Growth

Infarct growth, defined as the difference between ASPECTS and ASPECTS-POST, occurred more frequently in CCE patients (CCE: *n* = 21 [80.8%], NCCE: *n* = 11 [42.3%], *p* = 0.004). In addition, infarct growth was more severe in CCE patients (median ASPECTS decrease 2, range 0–10, IQR 2.25) than in NCCE patients (median ASPECTS decrease 0, range 0–9, IQR 1.5, *p* = 0.005).

### 3.7. Periprocedural Complications and In-Hospital Mortality

**Figure 2:**
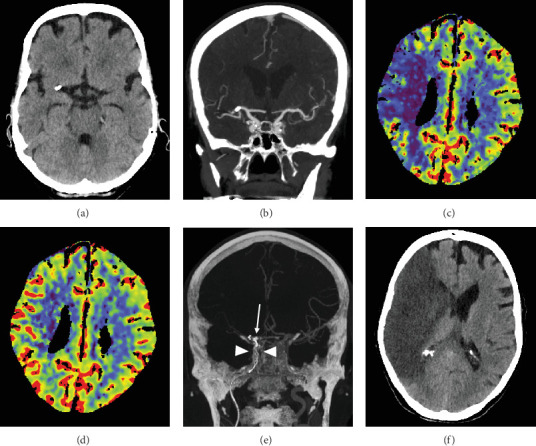
Exemplary case with periprocedural complication. (a, b) Calcified embolus in the right M1 segment, causing critical ischemia in the right MCA territory. The (c) CBF and (d) CBV CT perfusion maps show a large penumbra. (e) During the fourth thrombectomy maneuver, the stent-retriever got stuck in the distal ICA (arrowheads). The calcified embolus is visible near the distal stent-retriever markers (arrow). (f) Follow-up CT revealed complete infarction of the right MCA territory. CBF, cerebral blood flow; CBV, cerebral blood volume; ICA, internal carotid artery; MCA, middle cerebral artery.

Periprocedural complications tended to occur more frequently in CCE (*n* = 5, 19.2%) than in NCCE (*n* = 1, 3.8%, *p* = 0.095). In the CCE group, two dissections of the ipsilateral internal carotid artery occurred, which had to be treated with stents. In another CCE case, the stent-retriever got stuck in the internal carotid artery (Figure 2). The pusher wire then had to be partially removed surgically at the level of the proximal cervical segment. The embolus of this case was the one with the highest density in this study (max. density 1582 HU). Subarachnoid hyperdensities on postinterventional CT scans occurred more often in CCE (42.3%, *n* = 11) than in NCCE (15.4%, *n* = 4, *p* = 0.032). These were mild in most cases. Moderate to severe hyperdensities occurred in two CCE cases and in one NCCE case (*p* = 0.5). In-hospital deaths were observed without significant difference in patients with both CCE (*n* = 6) and NCCE (*n* = 4, *p* = 0.482).

Tables 1 and 2 provide a detailed overview of the results for CCE and NCCE patients (Tables 1 and 2).

## 4. Discussion

In this study, we analyzed the technical and clinical success of EVT in CCE, compared with NCCE. With a prevalence of 2.6%, CCE were rare in our patient cohort, which is consistent with previous works (prevalence 0.13%–1.8%) [8–12]. We observed longer procedure durations in CCE cases. Also, more EVT maneuvers were required to achieve the final recanalization result in CCE. For the first time, we compared the rate of FPR in CCE with a matched control group and found it to be lower in the CCE group. With 62%, successful recanalization was achieved less frequently for CCE patients compared with NCCE patients. Regarding clinical success, there was a lower NIHSS improvement after EVT in CCE. The mortality was not significantly higher in CCE patients. To our knowledge, this study is the first to investigate infarct growth after EVT of CCE compared with NCCE. Here, we found a more frequent occurrence of infarct growth in CCE. Also, the extent of infarct growth was substantially more pronounced than in NCCE. This finding helps to explain the poorer clinical results in CCE patients after EVT, as infarct growth has been found to influence functional outcome [18].

To date, there is little literature on EVT of CCE, but the number of studies on this subject has increased in recent years. The first case of thrombectomy of CCE was reported in 2016 by O'Cearbhaill et al., in which six stent-retriever maneuvers were required for a good angiographic result, but with only minimal clinical improvement [19]. Further case reports followed, including a report on the open neurosurgical recanalization of a middle cerebral artery, which underlines the special nature of this type of embolus [20]. The first original research on thrombectomy of CCE was performed by Koh et al. in 2017, with five CCE cases analyzed in a single-center study [8]. From 2018 to 2022, there were four multicenter studies with larger case numbers (*n* = 8–55) [9–12]. To our knowledge, the present work is the largest single-center study on EVT of CCE to date.

In terms of the technical success of EVT for CCE, our results are similar to previous studies. Maurer et al. and Dobrocky et al. also reported relatively low FPR rates (30% and 12.5%, respectively). In line with our results, low rates of successful recanalization (0%–57%) were also found in the other studies [8–12]. As in our study with a median of 77 min, long procedure durations for EVT of CCE were also found in earlier works (Bruggeman et al.: median 77 min; Grand et al.: mean 82 min; Maurer et al.: mean 93 min) [10–12]. Only Koh et al. reported lower procedure durations (median 30 min) [8]. However, in that study, only the manual aspiration technique was used (with a success rate of 0%), whereas in all other studies, including ours, stent-retriever-based EVT was predominantly performed. In an in vitro study with CCE analogs, contact aspiration also showed the lowest success rate [21]. Interestingly, the most successful approach in that work was the fixation of the CCE analogs between a stent-retriever and the tip of a balloon guide catheter with the removal of the entire device system under dual aspiration. This suggests that in addition to the mere choice of devices, the particular type of thrombectomy technique may be crucial for the success of recanalization in CCE.

Regarding clinical results following EVT of CCE, the literature to date indicates predominantly poor results, which is consistent with our findings [9–12]. In our study, about two-thirds of CCE patients were women, similar to most previous studies, which report 62%–69% [9–12]. Since poorer outcomes were reported for women after EVT in general, this could also impact the results after EVT of CCE [22–24]. Another cause could also be a higher age in CCE patients, which was observed both by us and by Bruggeman et al. [11].

Regarding periprocedural complications, we only observed a trend towards an increased occurrence in CCE. Complications reported in connection with CCE include ICA dissections, inadvertent stent-retriever detachment, breakage of the pusher wire, and vessel perforations with intracranial hemorrhage [9–12]. In addition to the hardness of CCE and the resulting increased risk of microwire perforation, it can be assumed that due to the high embolus stiffness, relatively strong traction forces are exerted on the cerebral vessels during EVT maneuvers, which is probably also the cause of the frequently observed subarachnoid hyperdensities on postinterventional CT scans in our study.

It is noticeable that different criteria were used for the detection of CCE in the mentioned studies. Like Maurer et al., we assumed calcified emboli at a maximum density value of at least 130 HU [10]. Bruggeman et al. defined a mean density of at least 90 HU as the cut-off value for the presence of CCE [11]. It is striking that Grand et al. assumed CCE already at a density of > 70 HU but found the lowest prevalence among all studies (0.13%) [12]. In our eyes, a cut-off value of 90 HU or less carries the risk of overestimating the presence of CCE, as subacute intracranial blood collections may show more than 80 HU [25]. In the studies by Koh et al. and Dobrocky et al., no exact criterion for the detection of CCE was specified [8, 9].  Table 3 provides an overview of the results of the present work and those of the previous studies (Table 3).

One of the limitations of our work is its retrospective design. Even though this is the largest single-center study to date to our knowledge, the number of cases is still small, which is due to the rarity of CCE. In our work, no histological characterization of the emboli was performed. In general, there is very little data on this so far. Only in the studies by Bruggemann et al. and Grand et al. were histological analyses available in 10% and 5% of cases, respectively [11, 12]. Furthermore, we were only able to assess the short-term clinical success based on the NIHSS data but had no data on the modified Rankin Scale or long-term follow-up.

## 5. Conclusion

Compared with NCCE, EVT for acute stroke due to CCE is associated with a lower technical success rate, including a lower frequency of FPR. Infarct growth occurs more frequently after EVT of CCE. Also, infarct growth is more pronounced in CCE than in NCCE. These findings help to explain the comparatively poor clinical results after EVT of CCE. In addition to the lower technical success rates, the age and sex of CCE patients probably also have an impact on the outcome. To improve the benefit of EVT in patients with CCE, devices specially tailored to this embolus type are desirable. Further research is needed to evaluate whether the use of next-generation devices such as stent-retrievers with closed distal ends or cage-like components can improve results [26–28]. Early and accurate detection of CCE in stroke imaging is crucial to select the appropriate EVT device from the start and may thus help to improve clinical outcomes. To determine the optimal cut-off value for the CT density of CCE, further studies with systematic histological analysis of the emboli are needed in this context.

## Figures and Tables

**Table 1:** Overview of CCE cases.

**CCE case no.**	**Sex**	**Age**	**Occluded segment**	**Embolus length (mm)**	**Max. embolus density (HU)**	**No. of maneuvers**	**Technique**	**Periprocedural complications**	**Final mTICI**	**Procedure duration (min)**	**ASPECTS**	**ASPECTS-POST**	**Intravenous thrombolysis**	**NIHSS (admission)**	**NIHSS (discharge)**
1	F	67	Right M2	13	230	2	2 x SRT	—	2b	78	8	8	+	14	13
2	F	69	Right M2	6	298	1	1 x SRT	—	2c	16	10	7	+	21	17
3	F	91	Left M2	4	724	2	1 x DAT, 1 x SRT	—	2c	88	10	10	−	11	9
4	F	83	Left M1	3	423	8	4 x DAT, 4 x SRT, rescue stenting of MCA	—	3	86	10	9	+	5	5
5	M	81	Left M2	2	151	2	2 x SRT	—	3	25	10	10	−	3	3
6	F	83	Left M1	4	292	5	5 x SRT	—	2b	88	10	8	−	15	20
7	F	74	Left terminal ICA	8	693	4	4 x SRT	—	3	47	10	10	+	18	14
8	F	83	Left M1	7	1073	2	2 x SRT	—	3	32	9	6	+	30	10
9	F	86	Left M2	4	333	4	4 x SRT	—	0	123	9	4	−	12	10
10	F	79	Right A3	6	346	7	7 x SRT	msHD	0	107	No infarcts in ACA territory	Subtotal infarction of ACA territory	−	4	24
11	F	73	Left M1	6	563	4	4 x SRT	—	2b	46	8	0	+	22	Deceased
12	M	89	Basilar artery	9	261	3	1 x DAT, 2 x SRT	—	2a	57	5 (pc-ASPECTS)	3 (pc-ASPECTS-POST)	−	28	Deceased
13	M	83	Right M2	5	718	1	1 x DAT	—	2c	76	10	9	+	16	14
14	M	47	Left M2	2	154	8	1 x DAT, 7 x SRT	—	2a	141	8	6	+	5	18
15	F	72	Right M1	8	1582	5	1 x DAT, 4 x SRT	The stent-retriever got stuck in the ICA	0	186	10	0	−	13	13
16	F	86	Left M1	29	172	3	1 x DAT, 2 x SRT	ICA dissection	2b	38	10	6	−	18	Deceased
17	F	69	Left terminal ICA	25	355	7	3 x DAT, 4 x SRT	msHD	2a	85	3	0	−	27	Deceased
18	M	82	Right M1	11	449	4	2 x DAT, 2 x SRT	—	2a	56	8	0	−	24	Deceased
19	M	79	Left terminal ICA	8	335	2	1 x DAT, 1 x SRT	—	3	109	10	10	−	2	0
20	F	76	Left M3	4	605	1	1 x SRT	—	1	36	10	8	−	5	3
21	F	83	Left M2	6	204	1	1 x SRT	—	2b	31	10	8	−	20	13
22	M	70	Right M1	9	1385	2	2 x SRT	—	3	63	10	9	−	7	5
23	F	75	Left A1	6	263	3	3 x SRT	—	0	145	Partial acute infarction of left ACA territory	New infarcts in left ACA territory	−	16	Deceased
24	F	77	Left M2	10	154	3	3 x SRT	ICA dissection	2b	79	7	6	−	12	5
25	M	80	Right M2	12	288	1	1 x SRT	—	2c	33	9	6	+	14	15
26	F	53	Right cavernous ICA	5	429	8	8 x SRT	—	0	230	9	3	−	9	32

Abbreviations: ACA, anterior cerebral artery; ASPECTS, Alberta Stroke Program Early CT Score; ASPECTS-POST, post-endovascular thrombectomy Alberta Stroke Program Early CT Score; CCEs, calcified cerebral emboli; DAT, direct aspiration thrombectomy; F, female; HU, Hounsfield units; ICA, internal carotid artery; M, male; MCA, middle cerebral artery; msHD, moderate to severe subarachnoid hyperdensities on postinterventional CT; mTICI, modified treatment in cerebral infarction score; NIHSS, National Institutes of Health Stroke Scale; pc-ASPECTS, posterior circulation Acute Stroke Prognosis Early CT Score; SRT, stent-retriever-based thrombectomy.

**Table 2:** Overview of matched NCCE cases.

**Matched NCCE case no.**	**Sex**	**Age**	**Occluded segment**	**Embolus length (mm)**	**Max. embolus density (HU)**	**No. of maneuvers**	**Technique**	**Periprocedural complications**	**Final mTICI**	**Procedure duration (min)**	**ASPECTS**	**ASPECTS-POST**	**Intravenous thrombolysis**	**NIHSS (admission)**	**NIHSS (discharge)**
1	F	67	Right M1	26	87	2	2 x DAT	—	2b	24	7	7	+	9	Deceased
2	F	69	Right M1	20	80	4	4 x SRT	—	2a	61	10	6	+	10	9
3	M	81	Left M2	12	76	1	1 x SRT	—	3	23	9	9	−	1	0
4	F	57	Left M1	15	85	1	1 x DAT	—	3	17	10	10	+	18	3
5	M	81	Left M2	27	73	1	1 x SRT	—	2b	28	10	8	−	18	16
6	F	83	Left M2	5	73	4	4 x SRT	msHD	2a	65	9	7	−	10	18
7	F	74	Right M2	8	80	1	1 x SRT	—	3	20	10	10	−	3	0
8	F	78	Left M1	8	77	1	1 x SRT	—	3	13	10	10	+	17	1
9	F	86	Left M1	11	79	2	2 x SRT	—	2b	46	6	5	+	17	16
10	F	82	Left A3	3	72	1	1 x SRT	—	3	34	No infarcts in ACA territory	No infarcts in ACA territory	−	13	11
11	F	73	Left M2	11	81	1	1 x SRT	—	3	11	9	8	−	8	6
12	F	87	Basilar artery	10	89	3	3 x SRT	—	2b	20	10 (pc-ASPECTS)	8 (pc-ASPECTS)	−	26	Deceased
13	M	70	Left M1	21	54	2	1 x DAT, 1 x SRT	—	2c	45	7	7	−	13	2
14	F	60	Left M2	8	78	1	1 x SRT	—	3	12	10	10	+	6	1
15	F	72	Right M2	6	54	2	1 x DAT, 1 x SRT	—	3	59	8	8	−	13	0
16	F	74	Right M1	18	57	4	2 x DAT, 2 x SRT	—	2c	88	7	7	+	16	3
17	F	77	Left M1	31	78	1	1 x DAT	—	3	26	10	9	−	22	14
18	M	82	Right M2	11	71	2	2 x SRT	—	2b	38	9	7	+	12	3
19	F	77	Left A1	10	75	1	1 x SRT	—	3	39	No infarcts in ACA territory	No infarcts in ACA territory	−	13	0
20	F	63	Left M1	19	83	2	1 x DAT, 1 x SRT	—	2b	63	9	0	+	19	4
21	F	83	Left M1	9	55	1	1 x DAT	—	3	24	10	9	−	18	1
22	M	87	Right M1	23	80	4	1 x DAT, 3 x SRT	—	3	35	10	10	+	18	Deceased
23	F	87	Left M2	5	56	1	1 x DAT	—	3	26	10	10	−	8	3
24	F	90	Right M1	4	57	4	3 x SRT, 1 x DAT	—	3	84	5	5	+	19	Deceased
25	M	80	Left M2	4	53	2	2 x SRT	—	2c	62	10	10	−	5	2
26	M	73	Left A2	7	59	1	1 x SRT	—	3	21	No infarcts in ACA territory	Partial infarction of left ACA territory	+	14	14

Abbreviations: ACA, anterior cerebral artery; ASPECTS, Alberta Stroke Program Early CT Score; ASPECTS-POST, post-endovascular thrombectomy Alberta Stroke Program Early CT Score; DAT, direct aspiration thrombectomy; F, female; HU, Hounsfield units; ICA, internal carotid artery; M, male; MCA, middle cerebral artery; msHD, moderate to severe subarachnoid hyperdensities on postinterventional CT; mTICI, modified treatment in cerebral infarction score; NCCEs, noncalcified cerebral emboli; NIHSS, National Institutes of Health Stroke Scale; pc-ASPECTS, posterior circulation Acute Stroke Prognosis Early CT Score; SRT, stent-retriever-based thrombectomy.

**Table 3:** Results of the present work and previous studies.

**Authors**	**Present study**	**Grand et al. [** 12 **]**	**Bruggeman et al. [** 11 **]**	**Maurer et al. [** 10 **]**	**Dobrocky et al. [** 9 **]**	**Koh et al. [** 8 **]**
No. of centers	1	37	16	7	2	1

No. of CCE cases	26	35	55	40	8	5

Total no. of patients	1004	27,394	3077	2969	639	“Approximately 450”

Prevalence of CCE	2.6%	0.13%	1.8%	1.3%	1.3%	1.1%

Definition of CCE	Max. density ≥ 130 HU	Density > 70 HU	Mean density ≥ 90 HU	Max. density ≥ 130 HU	Not specified	Not specified

Age (years)	CCE: 79 (median)	76 (mean)	CCE: 76 (median)	78 (mean)	80 (mean)	74 (median)
	NCCE: 73 (median)		NCCE: 72 (median)			
	*p* = 0.027		*p* = 0.004			

Female patients	CCE: 69%	66%	CCE: 62%	68%	63%	40%
	NCCE: 50%		NCCE: 48%			
	*p* = 0.054		*p* = 0.04			

No. of maneuvers	CCE: 3 (median)	3.3 (mean)	CCE: Median 2, IQR 1–4	2.7 (mean)	2.9 (mean)	Not reported
	NCCE: 1.5 (median)		NCCE: Median 2, IQR 1–3			
	*p* = 0.004		*p* = 0.21			

No. of FPR	CCE: 3 (11.5%)	Not reported	Not reported	12 (30%)	1 (12.5%)	0%
	NCCE: 12 (46.2%)					
	*p* = 0.006					

Procedure duration (min)	CCE: 77 (median)	82 (mean)	CCE: 75 (median)	93 (mean)	Not reported	30 (median)
	NCCE: 33 (median)		NCCE: 58 (median)			
	*p* < 0.001		*p* = 0.03			

Successful recanalization	CCE: 62%		CCE: 24/55 (43.6%)			0%
	NCCE: 92%	57%	NCCE: 1833/3022 (60.1%)	23/40 (57.5%)	1/8 (12.5%)	
	*p* = 0.009	(mTICI ≥ 2b)	aOR 0.52, 95% CI 0.28–0.97	(mTICI ≥ 2b)	(TICI ≥ 2b)	
	(mTICI ≥ 2b)		(eTICI ≥ 2b)			

NIHSS improvement	CCE: Median 2, range −23 to 20, IQR 2.75	Decrease of median NIHSS from 15 (admission) to 8 (discharge)	CCE: Median 2	Not reported	Not reported	Decrease of median NIHSS from 9 (admission) to 4 (discharge)
	NCCE: Median 5, range −8 to 17, IQR 11		NCCE: Median 4			
	*p* = 0.008		*p* = 0.008			

Infarct growth	CCE: Median ASPECTS decrease = 2	Not reported	Not reported	Decrease of median ASPECTS from 9 (initial) to 5 (after 24 h)	Not reported	Not reported
	NCCE: Median ASPECTS decrease = 0					
	*p* = 0.005					

Periprocedural complications	CCE: 19.2%	11.4%	CCE: 10.9%	10%	25%	0%
	NCCE: 3.8%		NCCE: 19.5%			
	*p* = 0.095					

Mortality	In-hospital mortality	Not reported	Mortality at 90 days			
	CCE: 23.1%		CCE: 35%	Mortality at 90 days	Mortality at 90 days	Mortality at 90 days
	NCCE: 15.4%		NCCE: 29%	56%	71%	0%
	*p* = 0.482		aOR 1.16, 95% CI 0.64–2.12			

Abbreviations: aOR, adjusted odds ratio; ASPECTS, Alberta Stroke Program Early CT Score; CCEs, calcified cerebral emboli; CI, confidence interval; eTICI, expanded treatment in cerebral infarction score; FPR, first-pass reperfusion; HU, Hounsfield units; IQR, interquartile range; mTICI, modified treatment in cerebral infarction score; NCCEs, noncalcified cerebral emboli; NIHSS, National Institutes of Health Stroke Scale.

## Data Availability

The data that support the findings of this study are available from the corresponding author upon reasonable request.
